# Are lateralized and bold fish optimistic or pessimistic?

**DOI:** 10.1007/s10071-024-01876-4

**Published:** 2024-06-04

**Authors:** F. Berlinghieri, G. Rizzuto, L. Kruizinga, B. Riedstra, TGG. Groothuis, C. Brown

**Affiliations:** 1 Groningen Institute for Evolutionary Life Sciences, University of Groningen, Groningen, 9747 AG The Netherlands; 2 Department of Biological Sciences, Macquarie University, North Ryde, NSW Australia; 3 CoNISMa, Consorzio Nazionale Interuniversitario per le Scienze del Mare, Rome, Italy

**Keywords:** Cognitive bias, Laterality, Boldness, Learning, Optimism versus pessimism, Fish

## Abstract

Cognitive bias is defined as the influence of emotions on cognitive processes. The concept of the cognitive judgement bias has its origins in human psychology but has been applied to animals over the past 2 decades. In this study we were interested in determining if laterality and personality traits, which are known to influence learning style, might also be correlated with a cognitive bias in the three-spined sticklebacks (*Gasterosteus aculeatus*). We used the judgement bias test with the go/no-go procedure where fish were first trained to discriminate between a black and white card and, after reaching a minimum learning criterion, tested their response to an ambiguous card (grey). Optimistic subjects were expected to have a high expectation of reward associated with an ambiguous stimulus, whereas pessimistic subjects a high expectation of non-reward. We used an emergence and a mirror test to quantify boldness and laterality, respectively. We hypothesised that male, bolder and more strongly lateralized fish would be more optimistic than female, shy and less strongly lateralised fish. We found that males and more strongly lateralized fish were more optimistic than females and less strongly lateralized fish. In addition, bold males were more optimistic than shy males as we predicted, but females showed the opposite pattern. Finally, fish trained on the black colour card learned the training task faster than those trained on a white card. Our results indicate that both laterality and personality traits are linked to animals’ internal states (pessimistic or optimistic outlooks) which likely has broad implications for understanding animal behaviour particularly in a welfare context.

## Introduction

The term cognitive bias is defined as the influence of emotions on cognition (Mathews and Macleod 1994). This occurs because the positive or negative valence of an affective state can influence cognitive processes such as perception and judgement of environmental input. (D’Ettorre et al. 2017; Novak et al. 2015). Harding et al. (2004) conveyed the concept of the cognitive judgement bias from human psychology to animals, determining the paradigm of the judgment bias test (Harding et al. 2004). Mendl et al. (2009) defined the cognitive judgement bias as “the propensity of a subject to show behaviour indicating anticipation of either relatively positive or relatively negative outcomes in response to affectively ambiguous stimuli” (Mendl et al. 2009 p. 164). Judgement bias is often analysed using a go/no-go procedure, where the animals are trained to discriminate two distinct stimuli and then, after reaching a minimum learning criteria, their response to an ambiguous stimulus is tested (Harding et al. 2004).

Individuals with positive cognitive judgement bias have a high expectation of reward when faced with an ambiguous stimulus (D’Ettorre et al. 2017). This state is commonly referred to as ‘optimism’ and operationally is observed when animals approach ambiguous stimulus quickly (Bateson and Nettle 2015; Roelofs et al. 2016). In contrast, individuals with negative cognitive judgment bias have low expectation of reward (or punishment) when confronted with an ambiguous stimulus. This state is commonly known as ‘pessimism’, and operationally is observed when animals either avoid ambiguous stimuli or approach it slowly (D’Ettorre et al. 2017; Douglas et al. 2012). In humans for instance, depressed people tend to react to ambiguous stimulus more negatively than people not affected by depression (Eysenck 1991; Richards et al. 2002, 2007).

While the concept of cognitive bias has its origins in human psychology, over the past two decades an increasing number of studies have been conducted in animal species using the animal’s judgement of an ambiguous stimulus, including fish (Espigares et al. 2022; Rogers et al. 2020), birds (Bateson & Matheson 2007; Brilot et al. 2010), and mammals (Bračić et al. 2022; Burman et al. 2011; Douglas et al. 2012). In European starlings (*Sturnus vulgaris*), for example, a judgmental cognitive bias test using the go/no-go task found a pessimistic bias in birds that have recently experienced a decline in environmental quality. During the training phase subjects had to distinguish between a white visual stimulus/palatable food and a dark grey stimulus/unpalatable food and later tested with the ambiguous stimulus (Bateson & Matheson 2007). Such tests offer unique insights into individuals subjective state and are increasingly used to assess animal welfare (Bateson & Matheson 2007). A systematic review and meta-analysis demonstrated that animals housed in good conditions are more likely to show optimistic judgements of an ambiguous stimulus compared to those in poor conditions (Lagisz et al. 2020).

Despite the link between welfare status cognitive bias test results, there is nonetheless considerable interindividual variation that remains largely unexplained. Here we focus on three potential sources of variability among individuals; personality, laterality and sex. Personality is described as individual differences in behaviour across time and context (Stamps and Groothuis 2010). Personality shapes how animals explore and interact with their environment, thereby influencing decision-making processes. Consequently, boldness, neophilia, activity and exploration could positively correlate with learning (Griffin et al. 2015). Indeed, animals that are more explorative, bold, and aggressive have been found to manifest a fast-learning style (Guido et al. 2017; Guillette et al. 2009; Mazza et al. 2018; Overington et al. 2011; Quinn et al. 2016; Tebbich et al. 2012). Bold individuals explore new objects and do so more willingly than a shyer conspecific and so establish new environmental contingencies quickly (Carazo et al. 2014; Raoult et al. 2017). In three spine-sticklebacks, for example, boldness affected information use, and consequently bold fish were better at perceiving and understanding environmental cues (Harcourt et al. 2010). In humans, certain personality traits have been found to correlate with cognitive processing of environmental stimuli, known as attention bias (Fulcher et al. 2008; Mathews et al. 1997). Personality makes some individuals more susceptible to developing attention bias during times of stress, and these biases can have positive feedback on personality traits (MacLeod et al. 2002). Thus, there is a theoretical connection between the response to an ambiguous stimulus and personality (D’Ettorre et al. 2017). Individuals could be inclined to develop positive or negative affective states relating to their personality traits, with consequent cognitive judgment bias (Mathews et al. 1997).

Cerebral lateralization, the asymmetrical processing of cognitive functions between the two brain hemispheres (Rogers 1989), has also been linked to variation in cognitive function. It has been shown that brain laterality improves cognitive abilities by dividing analyses of different types of information into the two cerebral hemispheres, allowing separate and parallel processing, thereby maximising processing efficiency (Rogers 2000). One advantage of having a lateralized brain is that it enables individuals to cope with two tasks at the same time (Dadda and Bisazza 2006). For example, an individual can forage and school with conspecifics simultaneously (Bibost and Brown 2013; Bisazza and Dadda 2005; Güntürkün et al. 2000). Having a lateralized brain can also be advantageous while learning. For instance, in goldbelly topminnows, *Girardinus falcatus*, strongly lateralized individuals were better than non-lateralized fish in a spatial learning task using geometrical cues (Sovrano et al. 2005). In birds, laterality can also influence problem solving. Strongly lateralized parrots performed better than less strongly lateralized individuals in pebble-seed discrimination test and a string-pull problem (Magat and Brown 2009). Lateralization also influenced the performance on spatial foraging assessment in captive parrots (*Amazona amazonica*) (Cussen and Mench 2014). However, to what extent lateralization is related to a cognitive bias is as yet unknown but given that the classic paradigm relies on a discrimination task, seems likely.

Sex differences in learning is widely reported in literature. In guppies, females displayed more behavioural flexibility than males, as they were more interested in problem solving during a novel foraging task (Laland and Reader 1999). Lucon-Xiccato and Bisazza (2017) found that female guppies solved a learning problem faster than males (Lucon-Xiccato and Bisazza 2017). However, this difference between the sexes can depend on the task (Etheredge et al. 2018). Indeed, in contrast to the previous cited studies, Fuss and Witte (2019) found that male Atlantic mollies learned the colour discrimination task faster than females. Success in solving various tasks may vary due to differences in life history. It is well established, for example, that females are highly motivated by food and males by sex, which relates to their varied life history priorities (Archard and Braithwaite 2011; Minter et al. 2017). It is reasonably well established that sex can also influence the outcome of cognitive bias tests. Lagisz et al., for example, conducted a meta-analysis of studies principally on mammals and found that housing manipulations were far more likely to have pronounced effect on judgment bias in males than females (Lagisz et al. 2020).

In the present study, we explored the possibility that sex, laterality and personality may correlate with cognitive bias since all traits are known to have strong impacts on cognition. We firstly analysed the behavioural laterality and boldness of sticklebacks, *Gasterosteus aculeatus*, and then performed a judgmental bias test using the classical go/no go paradigm followed by an ambiguous stimuli probe test to determine pessimism or optimism. We aimed to determine if a cognitive bias correlated with laterality, boldness and sex. Based on previous literature, we hypothesised that bolder and more strongly lateralized fish would be more optimistic (approach the ambiguous stimuli faster) than shyer, less-lateralised fish. Given that males tend to be less risk averse than females, we also predicted that males would be more optimistic than females.

## Methods

### Subjects

We used adult stickleback fish derived from wild marine migrants. These fish were previously used in a parental effects study (Berlinghieri et al. unpublished) in which only the parents were treated with perceived predation in a semi-natural environment and the offspring were tested for personality and laterality at 3 months of age. Here we test these offspring from this experiment at 1 year of age. Analyses revealed that there was no evidence of parental effects in laterality and boldness at this age (*p* > 0.275), thus, we did not include parental treatment in our analysis and fish from different parental treatments were pooled.

### Housing and experimental design

Fish were housed in a climate-controlled room in the animal facility of the University of Groningen. In order to be able to keep track of individual identity, fish were housed individually in a tank (30 × 16 × 18 cm), containing a plastic plant, PVC tubes for hiding, and a gravel substrate. All the tanks were connected to a recirculating water system at 17◦ (± 1) C. The photoperiod was set at 14:10 (L:D). Water parameters were continuously checked from the computer system and daily by animal caretakers. Fish were fed bloodworm and artemia once per day before the experiments and only with the reward (bloodworms) twice a day during the training phase for the cognitive bias test.

### Protocol

Fish were tested for personality and laterality using the emergence test as detailed below. The day after the first emergence test, fish started the judgement bias protocol. To determine repeatability of personality, the emergence tests was conducted a second time at the end of the judgement bias probe trials. Note we did not score laterality during the second trail.

### Emergence test

**Fig. 1:**
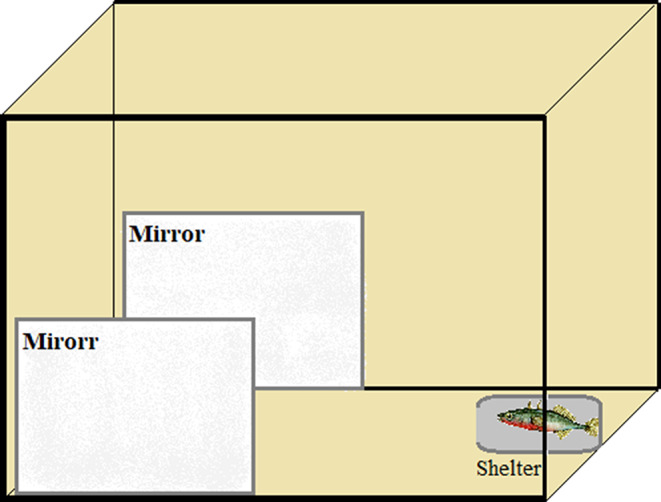
Set up for the emergence test. Individuals had to emerge from the shelter (boldness) and then approach one of two mirrors on either side of the tank to score their laterality

We conducted an emergence test to assess both boldness and laterality, eliminating the need to handle the fish in the interim. The experimental tank (40 × 30 × 40 cm) was positioned in an illuminated wooden box to avoid external disturbance. The tank had a gravel substrate, two mirrors placed on opposite sides of the tank at the far end and a shelter (10 × 10 × 10 cm) (Fig. 1). Each fish was gently placed inside the start box (shelter) which was then closed for 2 min. We remotely opened the box and the latency to emerge (the whole head had to be out of the shelter) was scored as a measure of boldness (Brown et al. 2007). Once the fish emerged and approached the two mirrors, their laterality was scored. Laterality scores were based on which eye they used to look at either mirror by pausing the video every 5s. Specifically, we scored the number of times they watched the mirror with their left, right, and both eyes and if they were not looking at the mirror based on body orientation (sensu Sovrano et al. 1999). Thus, our score of laterality is in a social context, but previous research suggests that laterality within individuals is reasonably consistent across contexts (Bisazza et al. 2001). The test lasted 15 min and was recorded using an overhead camera. Camera was an action Cam HDR-AS50 Sony. The latency to emerge and laterality were assessed using the software BORIS 7.10.2 (Friard and Gamba 2016). The sample size was 62 fish (30 females and 32 males).

### Cognitive judgement bias test

Fish were trained in their home tank (30 × 16 × 18 cm). The cognitive judgment bias test was divided into two phases: training and probe trails. We used back and white colour cards during the training phase and grey for the probe test (card dimensions: 5 × 12 cm, see Table 1 for card visual properties).

**Table 1:** Hue, chroma and brightness of the coloured cards used during the training phase and probe test measured by a spectrophotometer in the range of 200–700 nm

Colour	Hue	Chroma	Brightness
White	344	1.18	94.62
Black	210	44.5	2.34
Grey	222.75	1.97	34.8

**Fig. 2:**
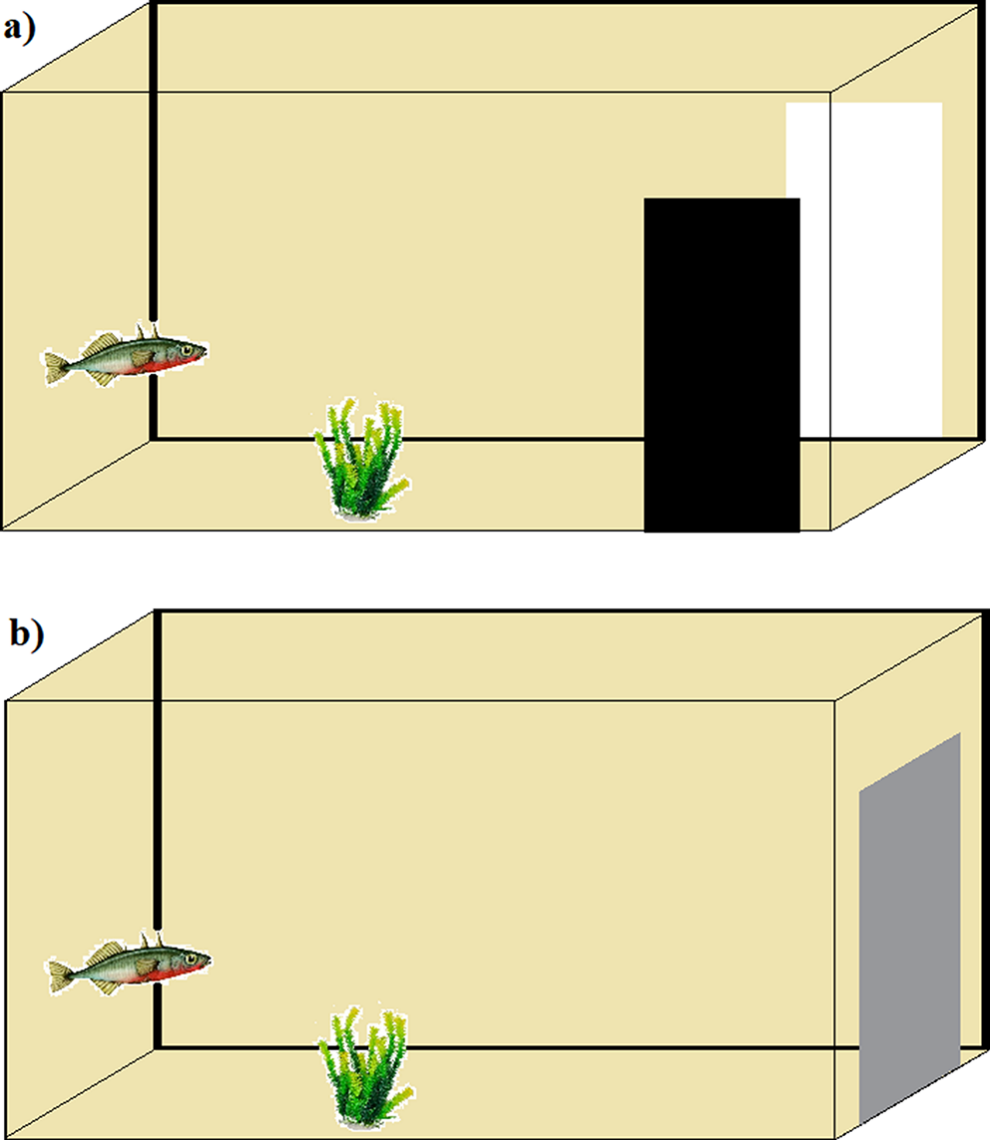
Training phase (**a**) with black or white cards placed on the left or right side of the testing arena. Probe test (**b**), time to approach the ambiguous stimulus, grey colour card placed in the centre of the tank. For a trial to commence, the subject had to be position in the centre of the far end of the arena. A food reward was delivered after the subject approached the correct card

During the training phase (Fig. 2a), half of the fish received a food reward (bloodworm) delivered by a plastic pipette if they approached a white card within one body length whereas if they approached a black card they were not rewarded (i.e. neutral outcome). For the remaining fish, the black card was rewarded and the white card was neutral. The position of the cards (left or right side) was fixed for each fish throughout the training phase to facilitate rapid training but varied between subjects. A brief pre-training phase of four trials over four days allowed the fish to become accustomed to the cards and the delivery of a food reward. After these pre-training trails, we started training and recorded if the fish went to the correct card or not (yes, no) and the latency to approach (seconds). A trial started when the fish was in the middle of the far end of the tank, the black and white cards were then simultaneously positioned on the sides of the tank (Fig. 2a). There were two training trials per day. Since the tanks were part of a recirculating water system, any potential odour cues from the food reward were quickly dispersed and because the food reward was offered only after the fish made their choice it was not possible for subjects to solve the task following odour plumes.

The training phase continued until the fish reached the minimum learning criteria or a maximum of 50 training trials whichever occurred sooner. To reach criteria, subjects had to select the rewarded card in 8 out of 10 trials. If the fish did not meet the minimum learning criteria within the 50 trials, it was excluded from further experimentation (3 males and 6 females).

The second phase of the experiments involved conducting three probe trials on three consecutive days where fish (*n* = 53) were exposed to an ambiguous cue. The first probe trial was conducted the day after the fish reached criteria, which was followed by a standard training trial. The second and the third probe trials were conducted on the following two days (i.e., one trial per day). The second probe trial was also followed by a standard training trial. During the probe trials, the subject was exposed to the ambiguous card (grey) in an ambiguous position (centre) (Fig. 2b). The time to approach the ambiguous stimulus was scored. If the fish failed to approach it was noted as 0 (failed) and a ceiling value (4 min) was entered for the latency data. Pessimistic fish were expected to avoid or approach the ambiguous symbol slowly, whereas optimistic fish should approach it quickly.

### Data analysis

We used the latency to emerge from the shelter during the emergence tests as our measure of boldness. Bold fish should emerge from shelter more quickly than shy fish (Brown et al. 2007). To assess the consistency of boldness scores across the two different trials, we estimated the adjusted repeatability with 1000 parametric bootstraps. We used the “rptR” package to calculate adjusted repeatability estimates for personality (RStudio 2022.07.2 + 576).

We calculated the strength of laterality based on eye use while looking at the mirror using the following formula:

$$Strenght \,of \,laterality=\left|\frac{\text{R}\text{i}\text{g}\text{h}\text{t} \,\text{e}\text{y}\text{e}-\text{L}\text{e}\text{f}\text{t} \,\text{e}\text{y}\text{e}}{\text{R}\text{i}\text{g}\text{h}\text{t} \,\text{e}\text{y}\text{e}+\text{L}\text{e}\text{f}\text{t} \,\text{e}\text{y}\text{e}}\right|$$

During the learning phase of the cognitive bias test, we used the number of trails to reach the learning criteria as a measure of learning speed. Since all fish approached the ambiguous stimuli during the test phase, we took the mean time to approach the ambiguous stimuli over the three probe trials as our measure of cognitive bias. Optimistic fish should approach more quickly than pessimistic fish.

The data from the probe trials did not follow a normal (Gaussian) distribution, being right skewed, so we employed Generalised Linear Models estimated using Maximum Likelihood. The response variables were modelled implementing a Gamma probability distribution with a log link function, which handles positively skewed data. We conducted two analyses. We first examined the mean time to approach the ambiguous stimuli during the three probe trials (mean Ct). Sex, boldness (from trial 1), and the strength of lateralisation (Laterality) were entered as independent variables. We also thought that their experience during the training phase might influence mean Ct, so training colour (black or white) and the number of trials to reach the learning criteria (Attempts) were also added to the model. It might be possible that fish that reached training criteria quickly might continue to do so in the probe trails (Mean Ct).

Response variables and predictors were first rescaled (subtracting the root mean square) to improve model convergence. We fitted different models according to our hypotheses and these were then compared using the Akaike Information Criterion (AIC), which penalises increasing model complexity to avoid over-fitting. Selection of the most parsimonious model was based on Delta AICc (AIC corrected for small sample size) ≥ 2 between the best model and the second-best model, and highest AIC weight (AICcWt), indicating the overall explanatory power among the set of models (see Tables 1 and 2). Since we built the models to test our hypotheses on selected variables from our training data set, we did not include the null model (intercept only) in the comparative analyses.

**Table 2:** MeanCT model selection based on AICc (akaike’s information criterion)

Model	K	AICc	Delta AICc	AICcWt	Cum.Wt	LL
Mod5	7	−4.12	0.00	0.97	0.97	10.43
Mod2	9	2.86	6.99	0.03	1.00	9.88
Mod1	7	10.65	14.78	0.00	1.00	3.04
Mod3	12	12.22	16.35	0.00	1.00	10.22
Mod4	8	13.52	17.65	0.00	1.00	3.04

Model: identifier of each model being tested; K: number of estimated parameters for each model; Delta AICc: difference between AIC score of each model and the best model; AICcWt: Akaike weights, indicates the level of support for each model; Cum.Wt: cumulative Akaike weights; LL: log-likelihood of each model

Modelling of the mean time to approach the ambiguous stimulus in the cognitive bias test started from the hypothesis of an interaction between Sex and Boldness, as they are often related, Laterality and Sex as sex differences in laterality are well known, and Laterality and card Colour to investigate a possible association between brain lateralization and colour preference (Karenina and Giljov 2022; Lopez-Persem et al. 2020). We also included a combination of Laterality, Attempts, and training Colour, to account for possible colour bias in the learning process and increase explanatory power (see Table 1). It seemed reasonable to assume that those individuals that approached the colour cards and learned the task quickly during the training phase might also continue that during the probe trials. Finally, we tested another potential interaction between Sex and Boldness, and Sex and Laterality, excluding training Colour and Attempts variables, with a reduction in model complexity. Note because the location of the coloured cards varied between individual fish, this is a true test of colour influence rather than being confounded by location. This led to five models being tested (the asterisk indicates an interaction in the following Models tested):

Mod1 “meanCT ∼ Sex + Boldness + Laterality + Attempts + Colour”

- Mod2 “meanCT ∼ Sex * Boldness + Sex + Boldness +Laterality + Attempts * Colour”
- Mod3 “meanCT ∼ Sex * Boldness + Sex + Boldness +Laterality * Attempts * Colour”
- Mod4 “meanCT ∼ Sex + Boldness + Attempts + Laterality * Colour”
- Mod5 “meanCT ∼ Sex + Boldness + Laterality + Sex*Boldness + Sex*Laterality”

Modelling of the number of attempts to reach learning criteria during the training phase was based on two possible interactions between predictor variables. In one scenario boldness, lateralisation and training colour interact to influence the number of attempts taken by a fish during training trials (Boldness * Laterality * Colour, interaction between boldness and laterality and training colour). Here, we were looking for a link between lateralization and boldness, which has been tested in other studies with diverse outcomes (Brown and Bibost 2014; Reddon and Hurd 2009). Alternatively, we expected an interaction between Sex and Boldness, and Laterality and training Colour (Sex * Boldness + Laterality * Colour), following the same criteria used in the models above. We also included the saturated model in our AIC test, which contrasted the simplest form, lacking interactions. This approach led to four models being tested:

- Mod1 “Attempts ∼ Sex + Boldness + Strength Laterality + Colour”
- Mod2 “Attempts ∼ Sex + Boldness * Strength Laterality * Colour”
- Mod3 “Attempts ∼ Sex * Boldness + Strength Laterality * Colour”
- Mod4 “Attempts ∼ Sex * Boldness * Strength Laterality * Colour”

To determine if certain fish simply approached any card quickly, we analysed the mean time to approach (MeanTimeApproach) the correct card (white or black depending on the fish) during the last five trials of the training phase. We tested the effect of Boldness, Laterality, Sex, Colour and their interactions. Data were log-transformed to improve model convergence. In order to account for all combinations between independent variables, we ran a multi-model inference “dredge” function (package MuMIn in R) and selected the best four performing models according the AICc criterion (see Table 8). As AICc scores were comparable, we retained the most informative model (model 4) which included two-way interactions between laterality and personality with the colour of the reward card.

- Mod1 “Time ∼ Boldness + 1”
- Mod2 “Time ∼ Boldness + Colour + Boldness: Colour + 1”
- Mod3 “Time ∼ Boldness + Laterality + 1”
- Mod4 “Time ∼ Boldness + Laterality + Colour + Boldness: Colour + Laterality: Colour + 1”

Lastly, we analysed the time to approach the white card and black card during the training phase, based on the last 5 trials per fish, and the grey card during the probe test using a Kruskal-Wallis test followed by pair-wise comparisons.

The training phase and probe test were executed by a singular author, whereas the emergence tests were collaboratively undertaken by two authors. However, video analysis was exclusively performed by a single author. Given that only one author assessed the videos in accordance with the established protocol for personality trait and laterality, the calculation of inter-rater reliability was not calculated.

## Results

Analysis of the time to emerge from shelter across the two trials indicated that this trait was repeatable (*R* = 0.308 ± 0.12, *P* = 0.009).

The best fitting model when examining the mean time to approach the ambiguous stimulus during the cognitive bias test was Mod5 (Table 3) which did not include any of the variables from the training period.

**Table 3:** Summary of GLM for meanCT

Predictors	MeanCT		
	Estimates	CI	*P*
(Intercept)	0.99	0.41 – 2.66	0.982
Sex [M]	0.09	0.02 – 0.36	**0.001**
Boldness	0.84	0.33 – 2.33	0.654
Laterality	0.37	0.15 – 0.96	**0.034**
Sex [M] × Boldness	23.99	3.53 – 187.24	**<0.001**
Sex [M] × Laterality	2.15	0.58 – 8.11	0.263
Observations	49		
R^2^ Nagelkerke	0.551		

**Fig. 3:**
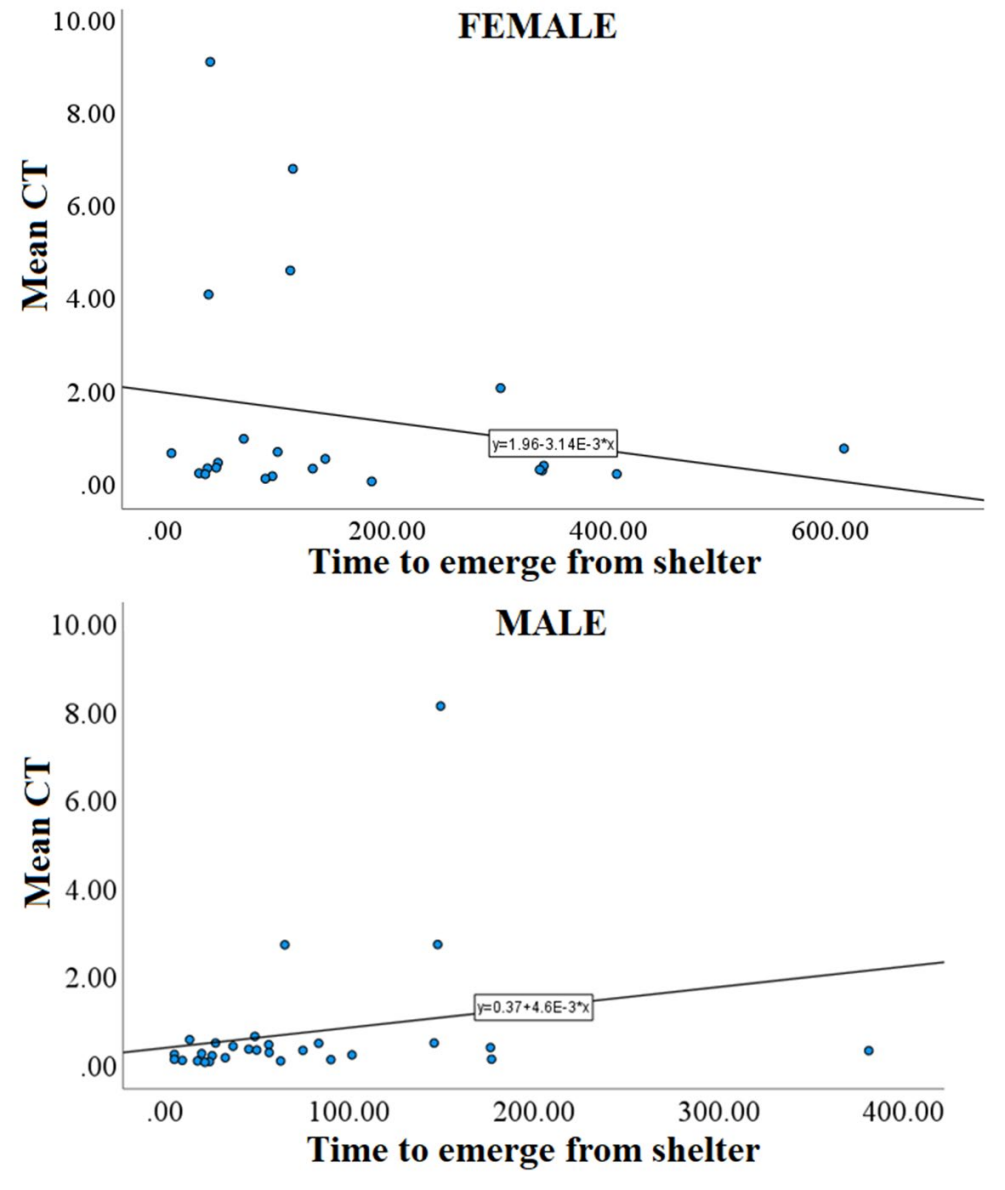
Mean time of three tests to approach the ambiguous stimuli during probe trials (MeanCT) as a function of the time to emerge from the shelter (Boldness) for females and males

**Fig. 4:**
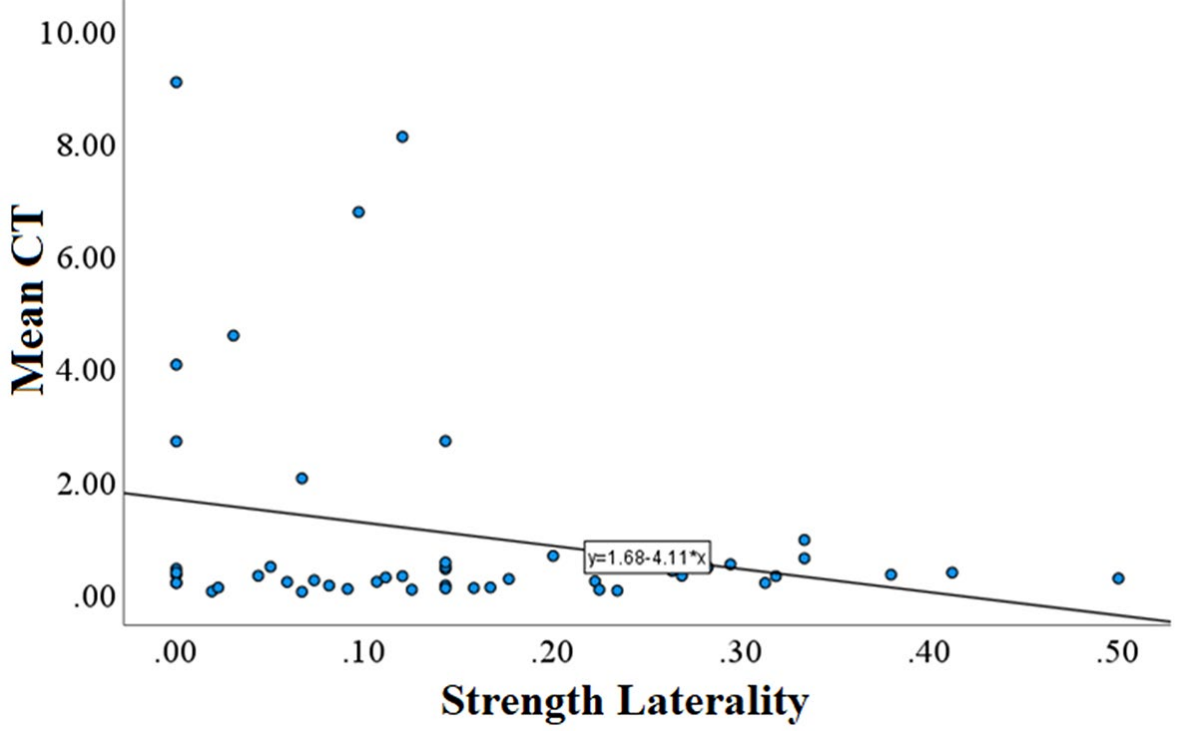
Strength of laterality with mean time to approach the ambiguous stimuli during probe trials (MeanCT). More strongly lateralised fish were faster to approach the ambiguous stimulus than less lateralised fish

The explanatory power of the model was substantial (Nagelkerke’s R2 = 0.551). By running the best model we found that sex had a significant effect on MeanCT with males being faster to approach the ambiguous stimulus than females (i.e. males more optimistic than females) (Table 2). The interaction between sex and boldness was also significant; females that exited the shelter faster (bolder) were slower to approach the ambiguous stimulus, whereas the opposite was true for males (Fig. 3). The effect of Laterality was also significant indicating more strongly lateralised fishes were faster to approach the ambiguous stimulus (Fig. 4; Table 2). We also analysed the possible correlation between laterality and boldness with Spearman correlation, but it was not significant (Males: *P* = 0.891, Spearman coefficient = 0.027; Females; *P* = 0.940, Spearman coefficient = 0.017).

When analysing the number of trials to reach criterion during the learning phase, the best fitting model was Mod1 (Table 4).

**Table 4:** Attempts model selection based on AICc

Model	K	AICc	Delta AICc	AICcWt	Cum.Wt	LL
Mod1	6	37.45	0	0.71	0.71	−11.74
Mod2	10	40.41	2.96		0.87	−7.31
Mod3	8	40.48	3.39	0.13	1	−10.62
Mod4	17	59.9	22.45	0	1	−3.08

**Fig. 5:**
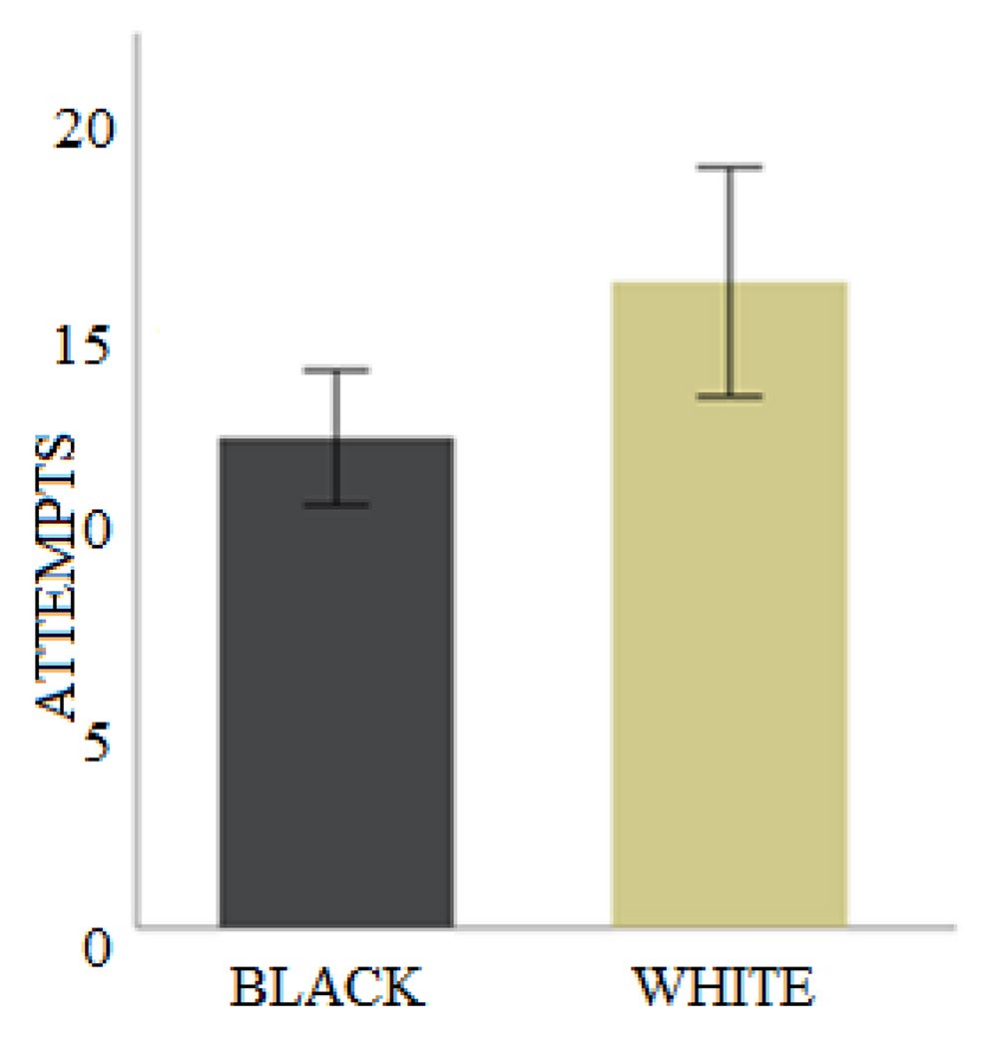
Mean and SE of the number of trials taken to reach the learning criterion during phase one (Attempts). Fish trained with a black card learned the task faster than those trained to approach the white card

The explanatory power of this model was relatively small (Nagelkerke’s R2 = 0.17). Nonetheless, the model captured a small positive effect of colour (Table 5) suggesting those fish trained to a black colour took fewer trials to learn the task than those trained to the white colour took (Fig. 5). Sex, boldness nor the strength of laterality impacted the number of trails to reach the criterion.

**Table 5:** Summary of GLM for attempts

Predictors	Attempts		
	Estimates	CI	*P*
(Intercept)	0.90	0.65 – 1.25	0.207
Sex [M]	0.89	0.68 – 1.16	0.386
Boldness	1.08	0.84 – 1.41	0.527
Laterality	0.89	0.70 – 1.14	0.240
Colour (W)	1.32	1.02 – 1.71	**0.034**
Observations	49		
R^2^ Nagelkerke	0.171		

When analysing the last five trials of the training phase, the best fitting model was Mod1 (Table 6). The explanatory power of this model was substantial (Nagelkerke’s R2 = 0.285). The model captured a positive effect on Boldness and the interaction between Boldness and Colour such that fish trained to the white card showed no effect of boldness, while those trained to black cards showed a slight positive relationship; Bolder fish approached the black card faster than shyer fish (R^2^ = 0.145; Table 7). Laterality had no significant effect on the time to approach the cards during training (Table 7).

**Table 6:** Time to approach model selection based on AICc

Model	Rank	Df.res	AIC	AICc	BIC	McFadden	Cox.and.Snell	Nagelkerke	*p*.value
Mod1	2	48	64.94	*−64.42*	*−59.20*	*−0.1295*	*0.1501*	*−0.05978*	0.3113
Mod2	4	46	*−*64.60	*−63.24*	*−55.04*	*−0.1879*	*0.2102*	*0.08369*	0.2417
Mod3	3	47	*−*64.03	*−63.14*	*48.42–56.39*	*−0.1470*	*0.6423 0.1686*	*−0.06711*	0.3255
Mod4	6	44	*−*65.12	*−62.45*	*−51.73*	*−0.2597*	*0.2784*	*−0.11080*	0.1107

**Table 7:** Summary of GLM for time to approach

Predictors	Time		
	Estimates	CI	*P*
(Intercept)	0.34	0.25 – 0.45	**<0.001**
Boldness	1.05	1.02 – 1.07	**<0.001**
Laterality	0.57	0.32 – 1.00	0.527
Colour (W)	1.12	0.71 – 1.77	0.637
Boldness × Colour (W)	0.96	0.92 – 1.00	**0.037**
Laterality × Colour (W)	2.07	0.91 – 4.73	0.091
Observations	50		
R^2^ Nagelkerke	0.285		

**Fig. 6:**
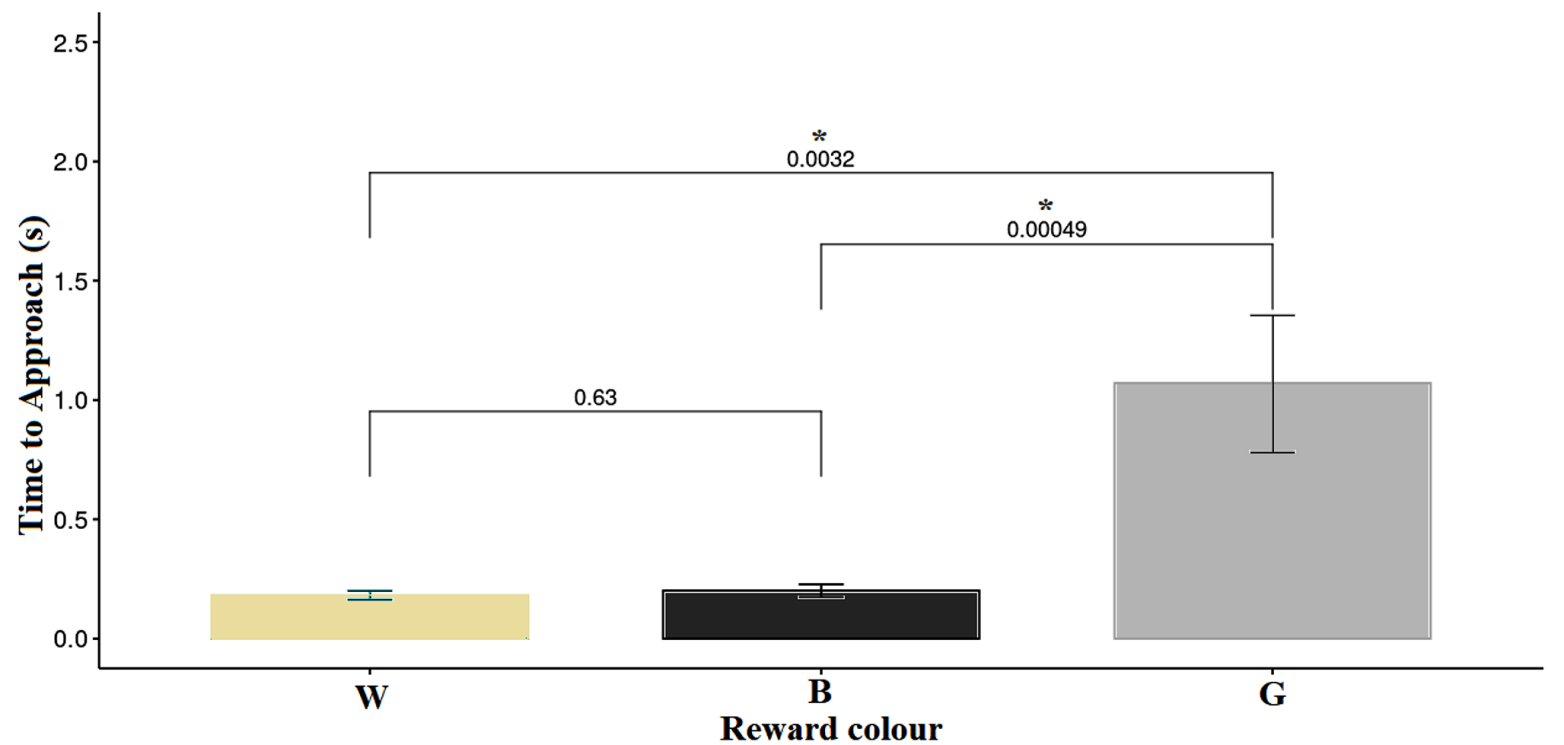
Comparison of the last five trials during the training phase with colours black or white and the probe test with the grey card

A comparison of the mean time to approach all three cards using Kruskal-Wallis test found a highly significant difference between cards (*P* < 0.001). Wilcoxon tests showed that the time to approach the ambiguous card (grey) was significantly longer than the time to approach either the wite or black card during training (*P* < 0.004 in both cases; Fig. 6).

## Discussion

The aim of the present study was to examine the potential effects of sex, laterality and personality on a cognitive judgement bias. Here we used the classic go/no go paradigm followed by an ambiguous stimuli probe test to determine pessimism or optimism. We found that the cognitive bias was influenced by the strength of laterality, sex, and the interaction between boldness and sex. As we hypothesised, more strongly lateralised fish were also more optimistic as evidenced by their faster approach to the ambiguous stimulus. We also found that males were more optimistic than females, but there was an interesting interaction between boldness and sex whereby bold males were more optimistic than shy males as we predicted, however females showed the opposite pattern. Finally, we found that sticklebacks trained to associate black cards with reward during the training phase took fewer trial to learn the task than those trained to a white card and fish took significantly longer to approach the ambiguous card during the probe trials.

Few researchers have considered how laterality may interact with cognitive bias. Here we found that more strongly lateralized fish as tested in a social context were faster to approach the ambiguous stimuli (i.e. were more optimistic) than less lateralized fish, suggesting a further possible explanation for the cognitive benefits of laterality: strongly lateralised fish are more likely to be optimistic and hence approached and interacted with novel objects, improving learning about new objects. While we only tested laterality in a social context in the present study (i.e., while looking at their reflection in a mirror), several studies have suggested that laterality tends to be consistent across multiple contexts (e.g., Bisazza et al. 2001). In addition, although laterality and boldness are sometimes correlated (Brown and Bibost 2014; Brown and Irving 2014; Reddon and Hurd 2009), we found no relationship between boldness and laterality in the present study. Interestingly, neither boldness nor laterality had an impact on the number of trials to learn the initial task during the training phase. However, when we analysed the average latency to approach the rewarded cards, there was an interesting interaction with boldness and card colour. Specifically, bolder fish approached the black card faster than shy fish, but we found no significant effect of the interaction between boldness and the white card. Collectively these results suggest that laterality and personality can influence both the training procedure and the ultimate outcome of cognitive bias tests using the go/no go procedure.

Sex is clearly an important predictor of affective states. A meta-analysis by Lagisz et al. which was dominated by studies on mammals, found that manipulations were far more likely to have pronounced effect on judgment bias in males than females (Lagisz et al. 2020). We found that male sticklebacks were generally more optimistic than females but this was tempered by boldness (see below). Interestingly sex had no influence on performance during the training phase. A study by Barker et al. (2016) examined how various housing cages (metabolic or standard housing) impacted the affective states of lab rats using the novel judgement bias paradigm (Barker et al. 2016). The testing context was very similar to the one we conducted herein (i.e., responding to a grey card as an ambiguous stimulus) and, in contrast with our results, they found that males housed in the metabolic cages exhibited a pessimistic judgement bias whereas females from those cages were more optimistic. Despite these contrasting outcomes, it is clear that sex can influence the outcome of judgement bias.

Our results are broadly in line with previous studies that show that males are generally less risk averse than females (Brown et al. 2005; Piyapong et al. 2010) which likely facilitates cognition because the subjects are more likely to explore and engage with the task. This outcome suggests that there may well be a link between personality traits such as boldness (tendency to take risks) and judgement bias. Indeed, we did find an interaction with boldness (which was highly repeatable) but the nature of that interaction varied with sex. One the one hand, males showed the predicted pattern in that bold males approached the ambiguous cue card more quickly (i.e., they were more optimistic) than shy males. Females, on the other hand, showed the opposite pattern, which is far more difficult to explain. Several studies in various species of mammals have identified links between personality traits and judgment bias. More social dogs, extraverted humans, proactive pigs, and less exploratory house mice all tend to be more optimistic (Asher et al. 2016; Barnard et al. 2018; Clegg 2018; Marshall et al. 1992; Verjat et al. 2021). In mound building mice, for example, Jardim et al. (2021) found that mice that had high exploration scores also explored the novel arm in a maze more, which although initially delayed their response, eventually resulted in faster approach latencies (Jardim et al. 2021). Personality traits are particularly well studied in fishes (Brown and Bibost 2014; Brown and Irving 2014; Colléter and Brown 2011) and we argue that they offer a great model for further exploring the relationship of personality traits with judgement bias (Espigares et al. 2022).

We also predicted that performance during the learning phase might influence the outcome of the probe trials in the test phase. Here we deliberately confounded card location and colour for each individual to facilitate rapid learning, which clearly worked given how quickly the fish reached learning criterion. During the probe trial the ambiguous card was intermediate both in colour and location. It seemed reasonable to assume that those individuals that approached the colour cards and learned the task quickly during the training phase might continue to do so during the probe trials. Contrary to our expectations, neither the colour the fish were trained to, nor the number of trials they took to reach the learning criteria influenced their optimism score. We did find, however, that the number of trails to reach the learning criterion was influenced by the colour of the positive reinforced card. Fish trained with black cards learned slightly faster than fish trained with the white card. This is likely due to the contrast between the card and the general background. As expected, the time taken to approach the ambiguous card was significantly slower than the time taken to approach either the black or white card during the training phase. This result clearly shows that the fish were confused by the unexpected card colour and location.

## Conclusion and implications

To our knowledge, this study is the first to evaluate the link between personality traits, laterality, and cognitive bias in fish. This is perhaps unsurprising since the vast majority of studies to date have focused on mammals, particularly in a welfare context (Lagisz et al. 2020). Nonetheless, given the rapid expansion of the aquaculture sector and growing public expectation of the use of welfare indicators in food production systems, it is imperative that we develop methods for investigating effective states in fishes. The finding that the interaction between personality traits and cognitive bias is shaped by the sex of the subject is particularly intriguing and deserves further study. Moreover, optimistic or pessimistic perceptions may have considerable, and as of yet unexplored, fitness implications for fishes (Espigares et al. 2022). The fact that the strength of laterality is positively related to the degree of optimism opens a new avenue for testing functional cognitive consequences of laterality.

## Data Availability

The authors declare that raw data are available at the data share repository Dryad with the identifier: DOI: 10.5061/dryad.1ns1rn920.
