# Correction to: Are lateralized and bold fish optimistic or pessimistic?

**DOI:** 10.1007/s10071-024-01894-2

**Published:** 2024-10-08

**Authors:** Flavia Berlinghieri, Gabriele Rizzuto, Lara Kruizinga, Bernd Riedstra, Ton G. G. Groothuis, Culum Brown

**Affiliations:** 1 Groningen Institute for Evolutionary Life Sciences, University of Groningen, 9747 AG Groningen, The Netherlands; 2 Department of Biological Sciences, Macquarie University, North Ryde, NSW Australia; 3 CoNISMa, Consorzio Nazionale Interuniversitario per le Scienze del Mare, Rome, Italy

**Correction to: Animal Cognition (2024) 27:42** 10.1007/s10071-024-01876-4

In this article few references were incorrectly published and should have been published as given below

Published as

Lopez-Persem A, Roumazeilles L, Folloni D, Marche K, Fouragnan EF, Khalighinejad N, Rushworth MFS, Sallet J (2020) Differential functional connectivity underlying asymmetric reward-related activity in human and nonhuman primates. Proc Natl Acad Sci USA 117(45):28452–28462. 10.1073/ PNAS.2000759117/SUPPL_FILE/PNAS.2000759117.SAPP. PDF

Should be published as

Lopez-Persem A, Roumazeilles L, Folloni D, Marche K, Fouragnan EF, Khalighinejad N, Rushworth MFS, Sallet J (2020) Differential functional connectivity underlying asymmetric reward-related activity in human and nonhuman primates. Proc Natl Acad Sci USA 117(45):28452–28462. 10.1073/pnas.2000759117

Published as

Lucon-Xiccato T, Bisazza A (2017) Individual differences in cognition among teleost fishes. In Behavioural Processes (Vol. 141, pp. 184– 195). Elsevier B.V. 10.1016/j.beproc.2017.01.015

Should be published as

Lucon-Xiccato T, Bisazza A (2017) Individual differences in cognition among teleost fishes. Behav Process 141(2):184–195. 10.1016/j.beproc.2017.01.015

Published as

Laland KN, Reader SM (1999) Foraging innovation in the guppy. Anim Behav (57)

Should be published as

Laland KN, Reader SM (1999) Foraging innovation in the guppy. Anim Behav 57(2):331–340. 10.1006/anbe.1998.0967

Published as

Lagisz M, Zidar J, Nakagawa S, Neville V, Sorato E, Paul ES, Bateson M, Mendl M, Løvlie H (2020) Optimism, pessimism and judgement bias in animals: a systematic review and meta-analysis. Neurosci Biobehavioral Reviews 118:3–17. 10.1016/J. NEUBIOREV.2020.07.012

Should be published as:

Lagisz M, Zidar J, Nakagawa S, Neville V, Sorato E, Paul ES, Bateson M, Mendl M, Løvlie H (2020) Optimism, pessimism and judgement bias in animals: a systematic review and meta-analysis. Neurosci Biobehav Rev 118:3–17. 10.1016/j.neubiorev.2020.07.012

Published as

Güntürkün O, Diekamp B, Manns M, Nottelmann F, Schwarz A, Skiba M (2000) Asymmetry pays: visual lateralization improves discrimination success in pigeons. In Current Biology (Vol. 10)

Should be published as:

Güntürkün O, Diekamp B, Manns M, Nottelmann F, Schwarz A, Skiba M (2000) Asymmetry pays: visual lateralization improves discrimination success in pigeons. Curr Biol 10(17):1079–1081. 10.1016/S0960-9822(00)00671-0

Published as

d’Ettorre P, Carere C, Demora L, Le Quinquis P, Signorotti L, Bovet D (2017) Individual differences in exploratory activity relate to cognitive judgement bias in carpenter ants. Behavioural Processes, 134, 63–69. 10.1016/j.beproc.2016.09.008

Should be published as:

d’Ettorre P, Carere C, Demora L, Le Quinquis P, Signorotti L, Bovet D (2017) Individual differences in exploratory activity relate to cognitive judgement bias in carpenter ants. Behav Process 134:63–69. 10.1016/j.beproc.2016.09.008

Published as:

Cussen VA, Mench JA (2014) Personality predicts cognitive bias in captive psittacines, Amazona amazonica. 10.1016/j. anbehav.2013.12.022

Should be published as:

Cussen VA, Mench JA (2014) Personality predicts cognitive bias in captive psittacines, *Amazona amazonica*. Anim Behav 89:123–130. 10.1016/j.anbehav.2013.12.022

Published as:

Clegg ILK (2018) Cognitive bias in zoo animals: An optimistic outlook for welfare assessment. In Animals (Vol. 8, Issue 7) MDPI AG

Should be published as:

Clegg ILK (2018) Cognitive bias in zoo animals: an optimistic outlook for welfare assessment. Animals 8(7):104. 10.3390/ani8070104 

The original article has been updated and published online.

